# Relationship between lower limb muscle activity and cortical activation among elderly people during walking: Effects of fast speed and cognitive dual task

**DOI:** 10.3389/fnagi.2022.1059563

**Published:** 2023-01-10

**Authors:** Jinuk Kim, Jungsoo Lee, Gihyoun Lee, Won Hyuk Chang, Myoung-Hwan Ko, Woo-Kyoung Yoo, Gyu-Ha Ryu, Yun-Hee Kim

**Affiliations:** ^1^Department of Health Sciences and Technology, Samsung Advanced Institute for Health Science and Technology, Sungkyunkwan University, Seoul, Republic of Korea; ^2^Ybrain Inc., Seongnam-si, Republic of Korea; ^3^Department of Medical IT Convergence Engineering, Kumoh National Institute of Technology, Gumi, Republic of Korea; ^4^Department of Physical and Rehabilitation Medicine, Center for Prevention and Rehabilitation, Heart Vascular Stroke Institute, Samsung Medical Center, Sungkyunkwan University School of Medicine, Seoul, Republic of Korea; ^5^Department of Physical Medicine and Rehabilitation, Jeonbuk National University Medical School, Jeonju, Republic of Korea; ^6^Department of Physical Medicine and Rehabilitation, Hallym University Sacred Heart Hospital, Anyang, Republic of Korea; ^7^Office of R&D Strategy and Planning, Samsung Medical Center, Seoul, Republic of Korea; ^8^Department of Medical Device Management and Research, Samsung Advanced Institute for Health Science and Technology, Sungkyunkwan University, Seoul, Republic of Korea; ^9^Department of Digital Health, Samsung Advanced Institute for Health Science and Technology, Sungkyunkwan University, Seoul, Republic of Korea

**Keywords:** elderly, gait, muscle activity, cortical activation, functional near-infrared spectroscopy, cognitive dual task

## Abstract

**Objective:**

Gait is a complex behavior that involves not only the musculoskeletal system, but also higher-order brain functions, including cognition. This study was performed to investigate the correlation between lower limb muscle activity and cortical activation during treadmill walking in two groups of elderly people: the young-old (aged 65–74 years) and the old-old (aged 75–84 years).

**Methods:**

Thirty-one young-old and 31 old-old people participated in this study. All participants were sequentially subjected to three gait conditions on a treadmill: (1) comfortable walking, (2) fast walking, and (3) cognitive dual-task walking. During treadmill walking, the activity of the lower limb muscles was measured using a surface electromyography system, and cortical activation was measured using a functional near-infrared spectroscopy system. The correlation between muscle activity and cortical activation during treadmill walking was analyzed and compared between the two groups.

**Results:**

During comfortable walking, lower extremity muscle activity had a strong correlation with cortical activation, especially in the swing phase; this was significantly stronger in the young-old than the old-old. During fast walking, the correlations between lower limb muscle activity and cortical activation were stronger than those during comfortable walking in both groups. In cognitive dual-task walking, cortical activation in the frontal region and motor area was increased, although the correlation between muscle activity and cortical activation was weaker than that during comfortable walking in both groups.

**Conclusion:**

The corticomotor correlation differed significantly between the old-old and the young-old. These results suggest that gait function is compensated by regulating corticomotor correlation as well as brain activity during walking in the elderly. These results could serve as a basis for developing gait training and fall prevention programs for the elderly.

## 1. Introduction

Walking performance declines with age (Sudarsky, 1990; Shimada et al., 2010). The gait pattern associated with aging is mainly characterized by decrease in gait speed, step length, and swing phase and increase in step width, double support time, and gait variability (Sudarsky, 1990). The decrease in walking ability that accompanies aging increases the risk of falls, decreases daily living activities and quality of life, and has a strong association with near-term mortality (Shimada et al., 2010; Studenski et al., 2011). Some elderly people change their gait patterns to compensate for reduced physical performance and reduce falls (Maki, 1997). These changes in gait patterns are particularly evident in the characteristics of muscle activity while walking. Elderly adults show a greater degree of activation of the tibialis anterior and soleus muscles than young adults during the loading response and mid-stance phases (Mickelborough et al., 2004). In addition, old adults have a higher degree of activation of the vastus lateralis and medial hamstring muscles during the loading response and mid-stance phases than do young adults, suggesting increased coactivation of the ankle and knee joint muscles (Schmitz et al., 2009).

Previous studies have investigated how age-related decline in gait capacity influences brain function (Harada et al., 2009) as gait is influenced by higher-order cognitive and cortical control mechanisms (Mihara et al., 2008). Central motor networks, including the primary motor (M1), premotor (PM), and prefrontal (PFC) cortices, are activated during walking (Mihara et al., 2008). Gait characteristics such as speed and variability are closely related to cortical activity that is regulated by multiple supraspinal control mechanisms (Wilson et al., 2019). In elderly people, slow gait speed is related to decline in the frontoparietal control network, which is associated with executive function, and gait stability is linked to the function of the dorsal attention network (Hsu et al., 2019). The gait control mechanism of the cranial nervous system is also affected by aging, and use of indirect motor pathways in the PM and supplementary motor areas and the PFC increases to maintain gait control despite age-related functional declines (Herold et al., 2018). Although studies have not measured real-time functional brain activity during actual walking tasks, they have delineated the neural correlates of gait *via* conventional neuroimaging tools, such as functional magnetic resonance imaging (fMRI) and positron emission tomography (PET). Combined functional near-infrared spectroscopy (fNIRS) and electroencephalography (EEG) is a recently developed imaging tool for measuring activities during walking or daily living activities (Gramigna et al., 2017; Jacobsen et al., 2021). Gait studies using fNIRS and EEG can help elucidate the characteristics of brain activity through real-time signal measurement (Khan et al., 2021).

Gait is a product of complex interactions between the central nervous and musculoskeletal systems (De Groote and Falisse, 1946). Simulations that predict movement patterns based on mathematical models of the neuro-musculoskeletal system can reveal the principles of gait movement by explaining causal relationships (Falisse et al., 2020). There is an increasing number of studies aiming to understand the cortical gait control mechanism based on experimental data. A study reported corticomuscular connectivity using EEG and electromyography (EMG) during treadmill walking as a novel technique based on reliable source localization and effective connectivity analysis. Those authors demonstrated that the motor cortex drives the movement of leg muscles through corticomuscular connectivity even during stereotypic locomotion (Artoni et al., 2017). These corticomuscular connections confirm cooperation between the cortex and muscles during walking, but additional research is needed to demonstrate how cortical activity relates to muscle activity. The relationships among aging, lower limb muscle activity, and cortical gait control mechanisms are also unclear. Therefore, investigation of age-related changes in cortical activities during gait using fNIRS will help elucidate the central mechanism of aging-induced decline of gait function in the elderly.

The decline in mobility among elderly people accelerates with age (Shumway-Cook et al., 2007). For example, people who are aged 75 years or older walk much more slowly with shorter steps and increased step width than people aged 65–74 years (Thaler-Kall et al., 2015). Therefore, geriatric studies often investigate the characteristics of aging by dividing the elderly population into two distinct groups; the young-old (65–74 years) and the old-old (75 years old and older; Magaziner, 1989). Walking ability and dual-task performance are lower in old-old people than in young-old people (Gimmon et al., 2018).

This study investigates the relationship between lower limb muscle activity and cortical activation in elderly people during walking and examines differences in that relationship between young-old and old-old subjects. This study also examines the effects of fast walking speed and cognitive dual-task walking on lower limb muscle activity, cortical activation, and the relationship between them in those two age groups.

## 2. Methods

### 2.1. Participants

Sixty-two healthy elderly people aged 65–84 years (26 males; mean age: 74.0 ± 4.8 years; range: 65–82 years) who met the inclusion criteria were enrolled in this study. They were divided into two groups based on age: the young-old group, aged 65–74 years (*n* = 31, 12 males; mean age: 70.0 ± 2.9 years) and the old-old group, aged 75–84 years (*n* = 31, 14 males; mean age: 78.0 ± 2.2 years; Magaziner, 1989). The number of participants was determined in accordance with the following criteria. Given that declining gait function in the elderly becomes evident in the age range of 75–80 years (Wilson et al., 2002), the participants were divided into two groups: the young-old (aged 65–74 years) and the old-old (aged 75–84 years; Fujii et al., 2020). The number of steps in 2 min, which is representative of gait function, differed between the young-old and the old-old. Specifically, the young-old and the old-old showed 90.77 ± 24.85 and 69.49 ± 37.88 steps in 2 min, respectively (Pothier et al., 2015). The effect size (0.664) was calculated using G*Power software to compare the differences between groups. Based on 0.8 power, 0.05 significance level, and 10% dropout rate, at least 31 participants were recruited in each group, for a total of 62 participants.

Written informed consent was obtained from all participants before the experiments, in accordance with the Declaration of Helsinki, and the protocol was approved by the Institutional Review Board of Samsung Medical Center (2020–09-172).

The inclusion criteria were (1) community-dwelling elderly people aged 65–84 years and (2) no history of central nervous system diseases. Participants were excluded if they (1) experienced difficulty in walking independently because of problems such as flatfoot, pes cavus, or visual field loss; (2) had a history of musculoskeletal disorders such as fracture or muscle or nerve injuries that could cause problems with lower extremity functions within 3 months before enrollment; (3) exhibited severe cognitive decline, with a score ≤ 10 on the Korean Mini-Mental State Exam (K-MMSE) (Na et al., 2006); had a serious mental illness, such as schizophrenia or bipolar disorder; or (4) were not appropriate for study participation. Prior to the experimental trial, information about sociodemographic characteristics (age, sex, and level of education), height, weight, body mass index, and medical history of all participants was obtained. The participants’ physical and gait functions were evaluated using the short physical performance battery (SPPB) (Guralnik et al., 1994), four-square step test (FSST) (Dite and Temple, 2002), timed up and go (TUG) test (Podsiadlo and Richardson, 1991), and 10-meter walking test (10MWT) (Collen et al., 1990). Cognitive function, depression, and daily living activities were examined using the K-MMSE (Lee et al., 2002), geriatric depression scale short form (GDS-SF) (Alden et al., 1989), and the EuroQol-5 dimension (EQ-5D) and Korean-modified Barthel index (K-MBI) (Jung et al., 2007), respectively.

### 2.2. Experimental protocol

According to the experimental protocol, each participant walked on a treadmill while their muscle activity and cortical activation were measured. All participants were sequentially subjected to three gait conditions on a treadmill: (1) walking comfortably at a self-selected speed, (2) walking fast with a motor load, and (3) walking while performing a cognitive task (i.e., with a cognitive load). The walking trials were arranged in a block paradigm. For each trial, the walking tasks involved a baseline standing state for 30 s before the trial and five task blocks (30 s in duration) alternating with five rest blocks (30 s in duration) for a total of 330 s. To prevent fatigue between conditions, the participants were allowed to rest for 5–10 min before each task block. The self-selected walking speed was that at which each subject felt comfortable walking on the treadmill, similar to their natural walking speed (Wass et al., 2005). The fast speed was 130% of the self-selected walking speed (Fukuchi et al., 2018). Before the measurement, participants were given time to adapt to both speeds and then allowed to rest. Grabbing the safety bar of the treadmill was permitted to prevent falls, but the subjects were instructed to exert as little force as possible along a line that did not affect walking. To minimize the influence of shoes, all participants wore running shoes of the same design. During the cognitive dual task, the participants were asked to walk while performing a phonemic verbal fluency task of saying as many words as possible, beginning with alternate letters as presented both aurally and visually (common consonants) (Dorfman et al., 2014). They were not specifically instructed to prioritize a task in the cognitive dual-task walking condition. Two letters were provided for every 30 s of walking task block to maintain a consistent level of cognitive effort during the task periods. Ten letters were randomly presented in all five blocks, and the total number of responses (words) spoken by each participant was recorded.

### 2.3. Experimental setup

#### 2.3.1. Surface electromyography

Muscle activity was synchronously recorded using a 12-channel surface electromyography (sEMG) system (TeleMyo Desktop DTS®, Noraxon, Scottsdale, AZ, United States). The sEMG system is a noninvasive method for measuring physiological muscle signals using electrodes attached to the skin surface above the muscles of interest. The sEMG signal was collected at a sample frequency of 1,000 Hz. The electrodes were positioned on the right rectus abdominus (RA), right erector spine (ES), bilateral hip flexor (HF), bilateral rectus femoris (RF), bilateral biceps femoris (BF), bilateral tibialis anterior (TA), and bilateral medial gastrocnemius (mGCM) muscles, as recommended by the Surface Electromyography for the Noninvasive Assessment of Muscles project (Merletti and Hermens, 2000). Switch sensors were placed on the surfaces of the big toes and heels of both feet to record the stance and swing phases during the gait cycle. Before the gait experiment, the maximum voluntary contraction (MVC) of the 12 muscles was recorded. Muscle activity during walking was normalized on the basis of the amplitude recorded during the measured MVC (Burden et al., 2003).

#### 2.3.2. Functional near-infrared spectroscopy

Cortical activation during treadmill walking was confirmed through oxyhemoglobin (oxyHb) and deoxyhemoglobin (deoxyHb) concentrations measured through fNIRS (NIRScout®; NIRx Medical Technology, Berlin, Germany). The fNIRS optodes comprised 22 LED light sources and 21 detectors, and 71 source-detector channels were used to monitor the hemodynamics. The topo map was the same as that used in a previous study (Kim et al., 2021). The regions of interest (ROIs) were the bilateral primary leg motor cortex (M1-leg, left: ch.61, right: ch.60), bilateral PM cortex (left: ch.41, ch.42, right: ch.33, ch.34), supplementary motor area (SMA, ch.9, ch.11), bilateral somatosensory motor cortex (S1, left: ch.44, ch.46, right: ch.37, ch.38), bilateral posterior parietal cortex (PPC, left: ch.65, ch.68, right: ch.66, ch.70), bilateral dorsolateral prefrontal cortex (dlPFC, left: ch.2, ch.6, right: ch.17, ch.19), and bilateral ventromedial prefrontal cortex (vmPFC, left: ch.5, ch.13, right: ch.17, ch.19). The fNIRS optodes were positioned in accordance with the international 10/20 system, and the channel distance (i.e., distance between the source and the detector) was 3.0 cm. The cranial vertex (Cz) beneath the first source was used as a marker to ensure replicable placement of optodes. After the Cz position was determined on each participant’s head, the fNIRS head cap was placed. For fNIRS, wavelengths of 760 and 850 nm were used with a sampling rate of 10.42 Hz.

### 2.4. Data preprocessing and analysis

#### 2.4.1. Surface electromyography

Muscle activity was measured and analyzed using Noraxon software (Myo Research XP Master Edition®, Noraxon). The measured sEMG signals were full-wave rectified, bandpass filtered (10–350 Hz), and normalized to the MVC amplitude values. These processed signals are highly related to actual muscle tension and strength and are used to estimate muscle activity (Clancy et al., 2006). Because use of the main muscles differs during the gait cycle, the activity of each of the 12 muscles was calculated as the average value across the eight selected stages of the gait cycle: initial contact (0–2% of the gait cycle), loading response (2–12%), mid-stance (12–31%), terminal stance (31–50%), pre-swing (50–62%), initial swing (62–75%), mid-swing (75–87%), and terminal swing (87–100%) (Hausdorff et al., 1995). The activity of HF, RF, BF, TA, and mGCM muscles was measured in both legs, and the average value was used for analysis.

The muscle coactivation index (CI) at each trunk, thigh, and shank part was calculated during walking. The CI is the ratio between the agonist and antagonist muscles in each of the trunk part (RA:ES), thigh part (RF:BF), and shank part (TA:mGCM) (Lee et al., 2017). It is used as a key indicator of joint stability during walking and to determine the degree of excessive energy consumption during walking (Hortobágyi et al., 2009). The CIs of the eight selected stages of the gait cycle were calculated using the following formula: antagonist activity was normalized to the mean gross muscle activity and multiplied by 2 to offset the activity of the agonist (Falconer, 1985).


$$CI=2\times \frac{sEMG_{antagonist}}{sEMG_{agonist}+sEMG_{antagonist}}\times 100\left(\%\right)$$

#### 2.4.2. Functional near-infrared spectroscopy

fNIRS data were processed using NIRS-SPM open-source software implemented in MATLAB® (MathWorks, Inc., Natick, MA, United States). In statistical parametric mapping analysis, a generalized linear model with standard hemodynamic response curves was established to simulate the hypothesized oxyHb response and examined to determine significant cortical activation during the experiment (Tak and Ye, 2014). At the group level, data were statistically analyzed on the basis of individual-level beta values to identify activated channels (corrected *p* < 0.05; Benjamini and Hochberg, 1995). Furthermore, the t-statistic maps computed for group analysis were plotted onto a conventional brain template, and regions with significant differences in oxyHb concentration were identified.

Changes in oxyHb concentration were analyzed using nirsLAB® (v.2019.04; NIRx Medical Technology, Berlin, Germany, NIRx Medical Technologies). Spike artifacts and discontinuous data in measurement signals were removed and replaced with the nearest signals. The raw data were bandpass filtered from 0.01 Hz to 0.2 Hz to remove baseline noise and eliminate possible respiration and heart rate signals (Robbins et al., 2012). Both oxyHb and deoxyHb signals were obtained during measurement, but only the oxyHb concentration was analyzed because of its superior signal-to-noise ratio (Herold et al., 2018). The oxyHb concentration for each of the 71 channels was calculated from preprocessed filtered data using the modified Beer–Lambert law (Strangman et al., 2002). For each channel, the grand average of each hemodynamic response was computed. Block averages for the 30-s walking trial periods were determined to extract integral values of changes in the oxyHb concentration. The representative integral values for each ROI were obtained from the average values of the channels included in that ROI. The coordinates and target ROIs were obtained using the fNIRS Optode Location Decider toolbox in MATLAB® (Zimeo Morais et al., 2018).

### 2.5. Statistical analysis

Data were statistically analyzed using IBM SPSS 20.0 (SPSS Inc., Chicago, IL, United States). Mean and standard deviation (mean ± SD) were calculated. Data normality and homogeneity of variances were confirmed with Shapiro–Wilk and Levene’s tests, respectively. Differences in demographic characteristics, function, treadmill speed, and verbal fluency responses between the young-old and old-old groups were investigated using independent t-tests and chi-square tests.

In addition, the %MVC in each muscle, CI in each joint muscle, and integral value of oxyHb in each ROI channel were compared using independent t-tests to confirm differences in cortical activation and muscle activity between the young-old and old-old groups under comfortable walking conditions. In all elderly subjects, the effects of fast speed and a cognitive dual task were compared using a paired t-test between experimental condition and comfortable walking. The interaction of groups and conditions was confirmed by conducting a 2 × 2 mixed-design analysis of variance (ANOVA) with fast speed or cognitive dual task as the independent within-subject variable and group (two levels: young-old and old-old) as the between-subject factor. When the condition × group interaction effect was significant, Bonferroni-adjusted *post hoc* tests were performed to analyze pairwise comparisons.

The relationship between muscle activity and cortical activation during gait was assessed using Pearson’s correlation coefficients. For lower extremity muscle activity, average %MVC and CI were used in the eight selected stages of the gait cycle. The average values of the HF, RF, BF, TA, and mGCM muscles measured on both sides were used. For cortical activation, the integral value of the average oxyHb concentration in the 30-s walking period in each ROI was used. Correlation coefficients between variables are indicated as correlation matrices in tables, and significant values are represented by a line between the cortex and muscle in the gait cycle. For all analyzes, the significance level was set at *p* = 0.05.

## 3. Results

### 3.1. Demographic and functional characteristics of the participants

Table 1 shows the characteristics of the participants and the differences between the young-old and old-old groups. The two groups differed significantly in age. Functionally, the SPPB, FSST, TUG, and 10MWT revealed that the old-old group had significantly lower physical and gait function than the young-old group (*p* < 0.05; Table 1). Furthermore, the K-MMSE, GDS-SF, and EQ-5D scores of the old-old group were significantly lower than those of the young-old group. No difference was observed in the K-MBI. While on the treadmill, the old-old group walked significantly more slowly than the young-old group at self-selected and fast walking speeds (*p* < 0.05; Table 1). The number of responses in the verbal fluency task during cognitive dual-task walking was significantly lower in the old-old group than in the young-old group (*p* < 0.05; Table 2).

**Table 1:** Demographic characteristics and functional differences between the young-old and old-old groups.

	Total (*n* = 62)	Young-old (*n* = 31)	Old-old (*n* = 31)	Value of *p*
Age (years, mean ± SD)	74.02 ± 4.81	69.97 ± 2.88	78.06 ± 2.21	**<0.001** ^*^
Sex (male: female)	26: 36	12: 19	14: 17	0.607
Height (cm, mean ± SD)	159.91 ± 8.50	161.24 ± 7.99	158.57 ± 8.90	0.110
Weight (kg, mean ± SD)	60.54 ± 9.84	61.65 ± 10.94	59.43 ± 8.65	0.190
BMI (kg/m^2^, mean ± SD)	23.74 ± 3.04	23.92 ± 3.15	23.56 ± 2.97	0.322
Education (*N* (%))
More than high school	37 (59.7)	21 (67.7)	16 (51.6)	0.191
Medical history (*N* (%))
Neck pain	4 (6.5)	3 (9.7)	1 (3.2)	0.301
Low back pain	11 (17.7)	6 (19.4)	5 (16.1)	0.740
Rheumatoid arthritis	2 (3.2)	1 (3.2)	1 (3.2)	1.000
Osteoarthritis	7 (11.3)	2 (6.5)	5 (16.1)	0.229
High blood pressure	33 (53.2)	14 (45.2)	19 (61.3)	0.203
Diabetes	10 (16.1)	4 (12.9)	6 (19.4)	0.490
Heart disease	5 (8.1)	1 (3.2)	4 (12.9)	0.162
Medication usage [*N* (%)]	18 (58.1)	23 (74.2)	41 (66.1)	0.180
SPPB (mean ± SD)	11.10 ± 1.17	11.61 ± 0.67	10.58 ± 1.34	**<0.001** ^*^
FSST (sec, mean ± SD)	8.25 ± 1.32	7.55 ± 1.21	8.95 ± 1.04	**<0.001** ^*^
TUG (sec, mean ± SD)	8.22 ± 1.34	7.64 ± 0.98	8.81 ± 1.42	**<0.001** ^*^
10MWT (m/s, mean ± SD)	1.40 ± 0.21	1.48 ± 0.17	1.32 ± 0.22	**<0.001** ^*^
K-MMSE (mean ± SD)	26.19 ± 3.27	26.94 ± 2.25	25.45 ± 3.94	**0.037** ^*^
K-MBI (mean ± SD)	99.97 ± 0.25	100.00 ± 0.00	99.94 ± 0.36	0.161
EQ-5D (mean ± SD)	0.89 ± 0.09	0.92 ± 0.07	0.86 ± 0.10	**0.003** ^*^
GDS-SF (mean ± SD)	3.32 ± 3.80	1.81 ± 2.61	4.85 ± 4.21	**<0.001** ^*^

SD, standard deviation; BMI, body mass index; SPPB, short physical performance battery; FSST, four square step test; TUG, timed up and go test; 10MWT, 10-meter walking test; K-MMSE, Korean Mini-Mental State Exam; K-MBI, Korean-modified Barthel index; EQ-5D, EuroQol-5 dimension; GSD-SF, geriatric depression scale short form; *bold font, significant difference between the young-old and old-old groups (*p* < 0.05).

**Table 2:** Treadmill speed and verbal fluency performance during dual-task walking in the young-old and old-old groups.

	Total (*n* = 62)	Young-old (*n* = 31)	Old-old (*n* = 31)	value of *p*
Treadmill speed (km/h, mean ± SD)
Self-selected walking	3.3 ± 0.5	3.5 ± 0.5	3.2 ± 0.5	**0.005** ^*^
Fast walking	4.3 ± 0.7	4.6 ± 0.6	4.1 ± 0.6	**0.007** ^*^
Verbal fluency performance (mean ± SD)	31.3 ± 11.4	36.7 ± 10.9	26.4 ± 9.6	**0.007** ^*^

SD, standard deviation; *bold font represents significant difference between the young-old and old-old groups (*p* < 0.05).

### 3.2. Muscle activity and cortical activation during comfortable walking

#### 3.2.1. Muscle activity

**Figure 1:**
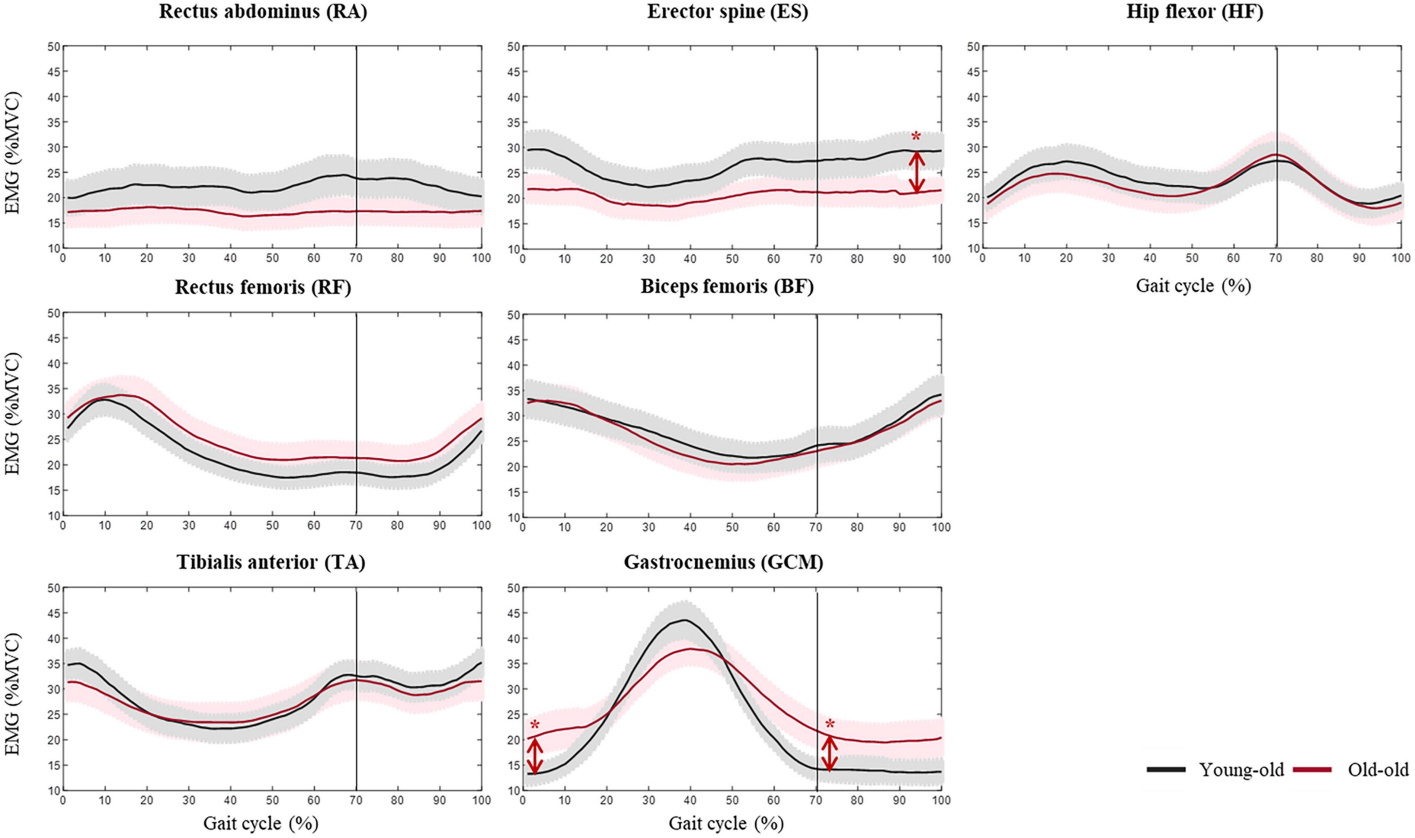
Lower limb muscle activity during comfortable walking in the young-old and old-old groups. *Significant difference between groups (*p* < 0.05).

Figure 1 shows the lower limb muscle activity patterns during comfortable walking in the young-old and old-old groups. The old-old group had greater mGCM muscle activity than the young-old group in the initial contact and initial swing phases (*p* < 0.05). However, the old-old group had lower ES muscle activity than the young-old group in the terminal swing phase (*p* < 0.05; Supplementary Table S1). The old-old group had a significantly higher CI of the ankle joint than the young-old group in the initial contact, loading response, initial swing, mid-swing, and terminal swing phases (*p* < 0.05; Supplementary Table S2).

#### 3.2.2. Cortical activation

**Figure 2:**
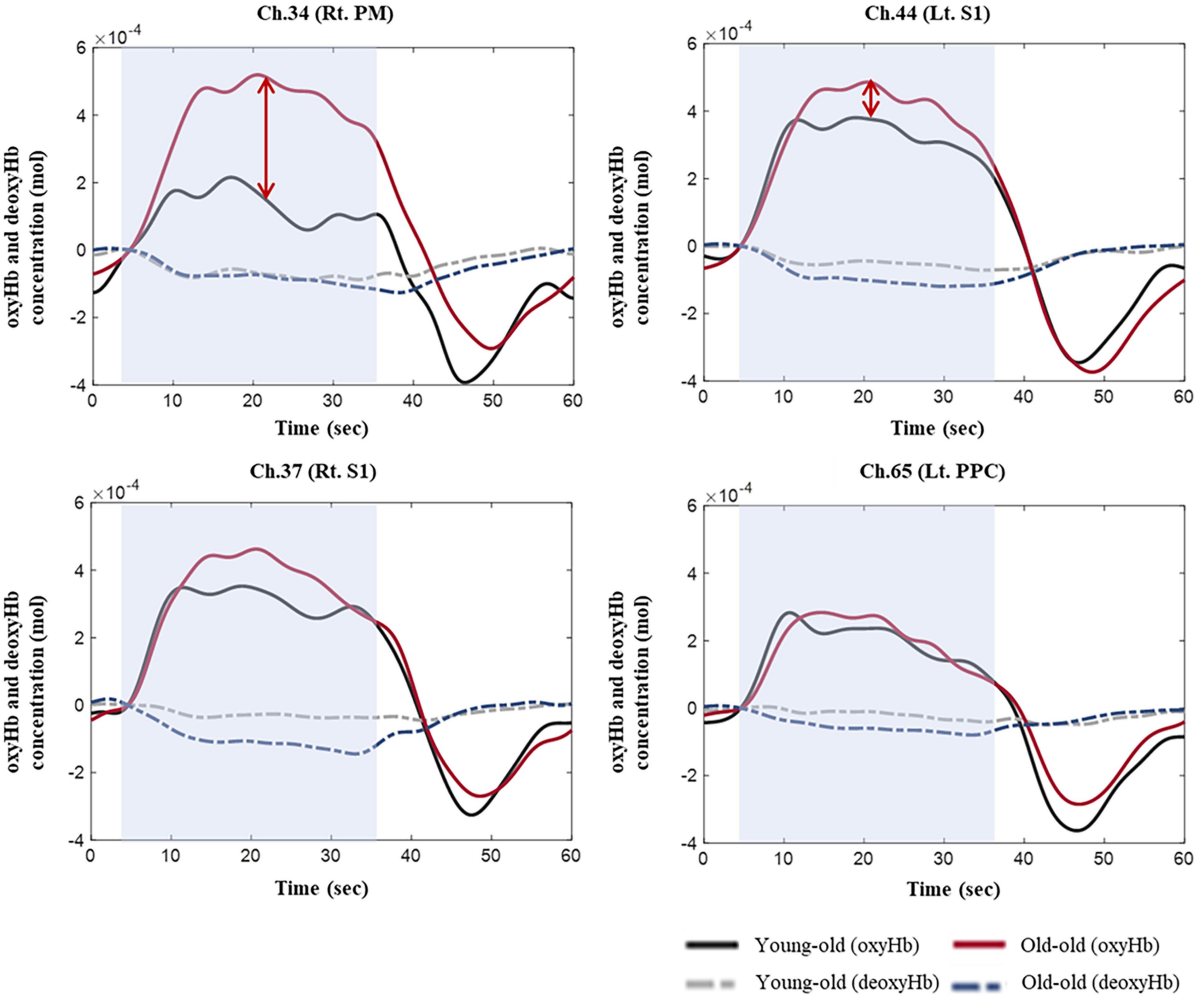
OxyHb and deoxyHb concentrations during comfortable walking in the young-old and old-old groups.

Supplementary Table S3 lists the integral value of oxyHb concentrations in each ROI during comfortable walking. No significant differences were observed between the young-old and old-old groups, but the old-old had a higher integral oxyHb concentration than the young-old (Supplementary Table S3; Figure 2).

#### 3.2.3. Relationship between lower limb muscle activity and cortical activation

**Figure 3:**
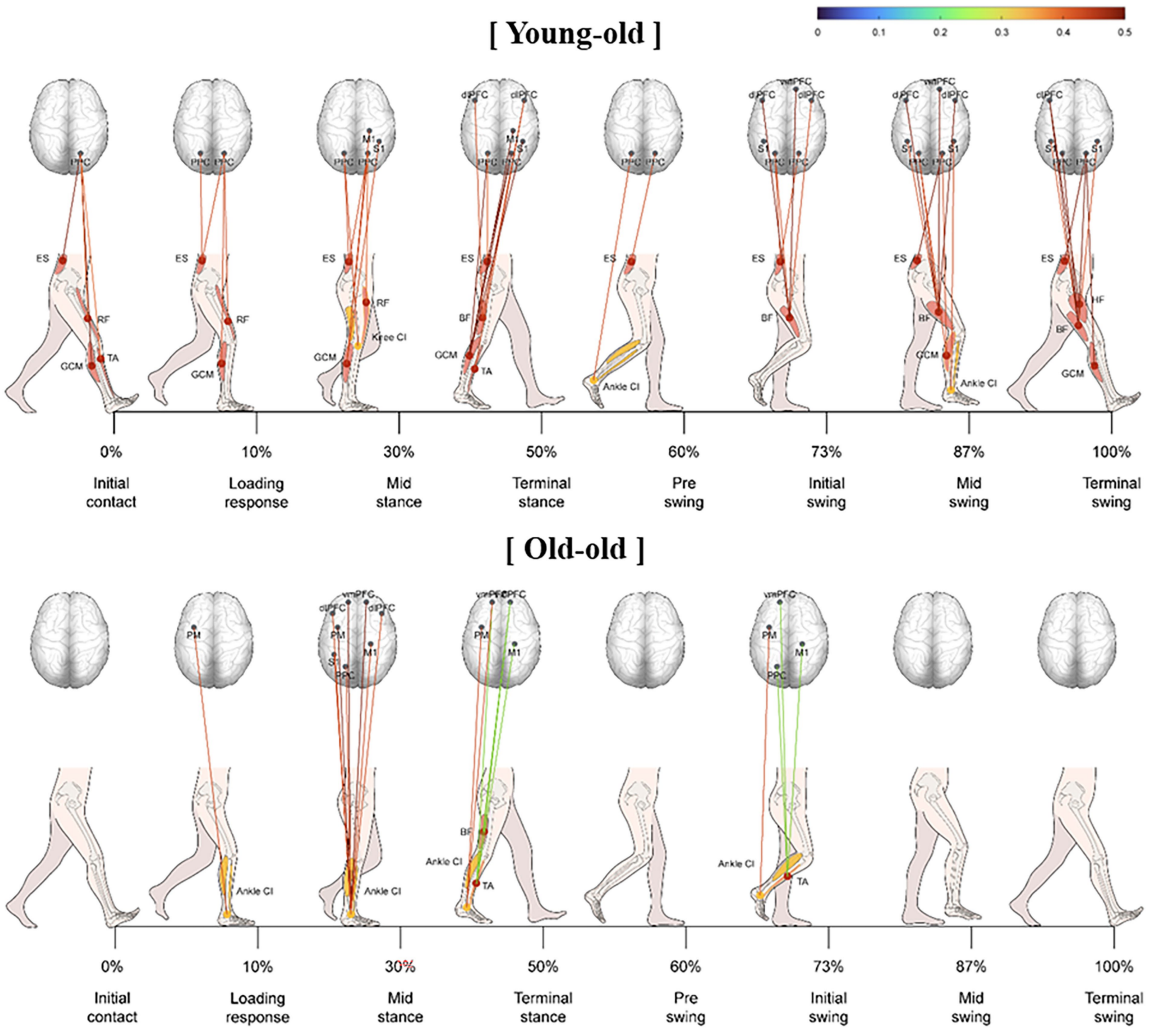
Significant correlation between lower limb muscle activity and cortical activation during comfortable walking in the young-old and old-old groups.

Figure 3 shows the significant correlations between muscle activity and cortical activation in elderly participants during comfortable walking. In the initial contact, loading response, mid-swing, and terminal swing phases, PPC activity correlated significantly with ES muscle activity. The CI of trunk, thigh, and shank parts had a significantly stronger positive correlation with oxyHb concentration in the bilateral PM, left S1, left PPC, and left dlPFC in the swing phase than in the stance stage (*p* < 0.05). These correlations were significantly stronger in the young-old group than that in the old-old group during comfortable walking (*p* < 0.05; Figure 3). In the young-old group, the oxyHb concentration in the sensorimotor region had a significant correlation with leg muscle activity throughout the gait cycle. In addition, the significant correlation in the swing phase was stronger than that in the stance phase, and a correlation with the frontal lobe was shown from the terminal stance to the terminal swing. In the old-old group, the correlation between oxyHb concentration in the sensorimotor region and leg muscle activity was significant only in mid-stance phase (*p* < 0.05; Figure 3).

### 3.3. Effects of fast speed

#### 3.3.1. Muscle activity

Supplementary Table S4 shows the comparison of lower limb muscle activity patterns during fast walking and comfortable walking among our elderly participants. All participants had significantly increased activity of the HF, RF, BF, TA, and GCM during fast walking compared with that during comfortable walking in the initial contact and loading response phases (*p* < 0.05). In the mid-stance phase, HF, BF, and GCM activity was greater during fast walking than during comfortable walking (*p* < 0.05). In the terminal stance, all participants had significantly increased HF and GCM activity during fast walking compared with that during comfortable walking (*p* < 0.05). Increased lower limb muscle activity during fast walking was also observed in the swing phase. The activities of the RA and BF muscles increased significantly during fast walking in the pre-swing phase (*p* < 0.05). RA muscle activity was likewise significantly increased in the initial swing phase during fast walking compared with that during comfortable walking (*p* < 0.05). In the mid-swing and terminal swing phases, all participants had significantly greater activity in the HF, RF, BF, and TA muscles during fast walking than during comfortable walking (*p* < 0.05). The whole study population also had significantly increased CI of the ankle joint during fast walking compared with that during comfortable walking in the initial contact and loading response phases (*p* < 0.05; Supplementary Table S5).

#### 3.3.2. Cortical activation

Supplementary Table S6 presents the integral value of oxyHb concentration in each ROI during fast walking. When walking fast, our elderly population exhibited significantly decreased integral values of oxyHb concentration in the SMA (ch.9) and left vmPFC (ch.5) compared with the values during comfortable walking (*p* < 0.05).

#### 3.3.3. Relationship between lower limb muscle activity and cortical activation

**Figure 4:**
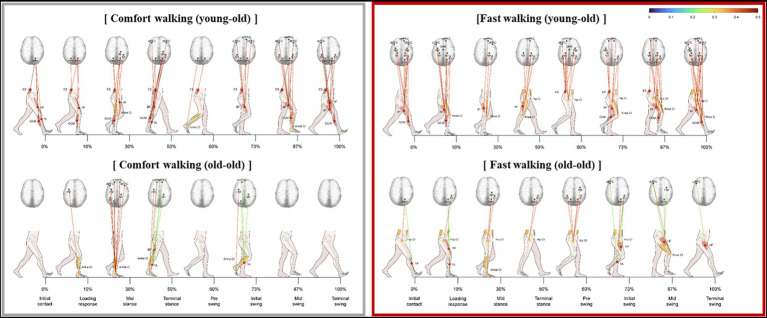
Significant correlation between lower limb muscle activity and cortical activation during fast walking in the young-old and old-old groups.

The significant correlation between lower limb muscle activity and cortical activation was stronger during fast walking than during comfortable walking in both groups (Figure 4). Hip muscle activity had a significantly stronger correlation with coactivation of the trunk during fast walking than during comfortable walking. In addition, the correlations between the HF, BF, and CI values of the trunk and thigh were stronger in the swing phase.

### 3.4. Effects of cognitive dual-task walking

#### 3.4.1. Muscle activity

Supplementary Table S7 shows the lower limb muscle activity patterns in our elderly study population during cognitive dual-task walking compared with those during comfortable walking. The whole population had significantly increased activity in the BF and TA muscles during cognitive dual-task walking compared with those during comfortable walking in the initial contact and loading response phases (*p* < 0.05). However, in the terminal stance phase, GCM activity decreased during cognitive dual-task walking (*p* < 0.05). In the pre-swing and initial swing phases, the whole study population had significantly increased activity of the RA muscles during cognitive dual-task walking compared with that during comfortable walking (*p* < 0.05). The activity of the RF muscle in the mid-swing phase and the BF muscle in the terminal swing phase increased during cognitive dual-task walking compared with those during comfortable walking. The participants had a significantly decreased CI of the knee joint during cognitive dual-task walking compared with that during comfortable walking in the pre-swing and initial swing phases (*p* < 0.05; Supplementary Table S8). In the mid-swing phase, the CI of the ankle joint decreased significantly during cognitive dual-task walking compared with that during comfortable walking.

#### 3.4.2. Cortical activation

Supplementary Table S9 describes the integral value of the oxyHb concentration during cognitive dual-task walking in each ROI. When the participants were walking while performing a cognitive task, the integral values of the oxyHb concentration in the bilateral M1-leg (ch.60, ch.61), bilateral PM (ch.41, ch.42, ch.33, ch.34), SMA (ch.9, ch.11), left S1 (ch.46), right PPC (ch.66, ch.70), bilateral dlPFC (ch.2, ch.6, ch.17, ch.19), and bilateral vmPFC (ch.5, ch.13, ch.15, ch.22) increased significantly compared with those during comfortable walking (*p* < 0.05).

#### 3.4.3. Relationship between lower limb muscle activity and cortical activation

**Figure 5:**
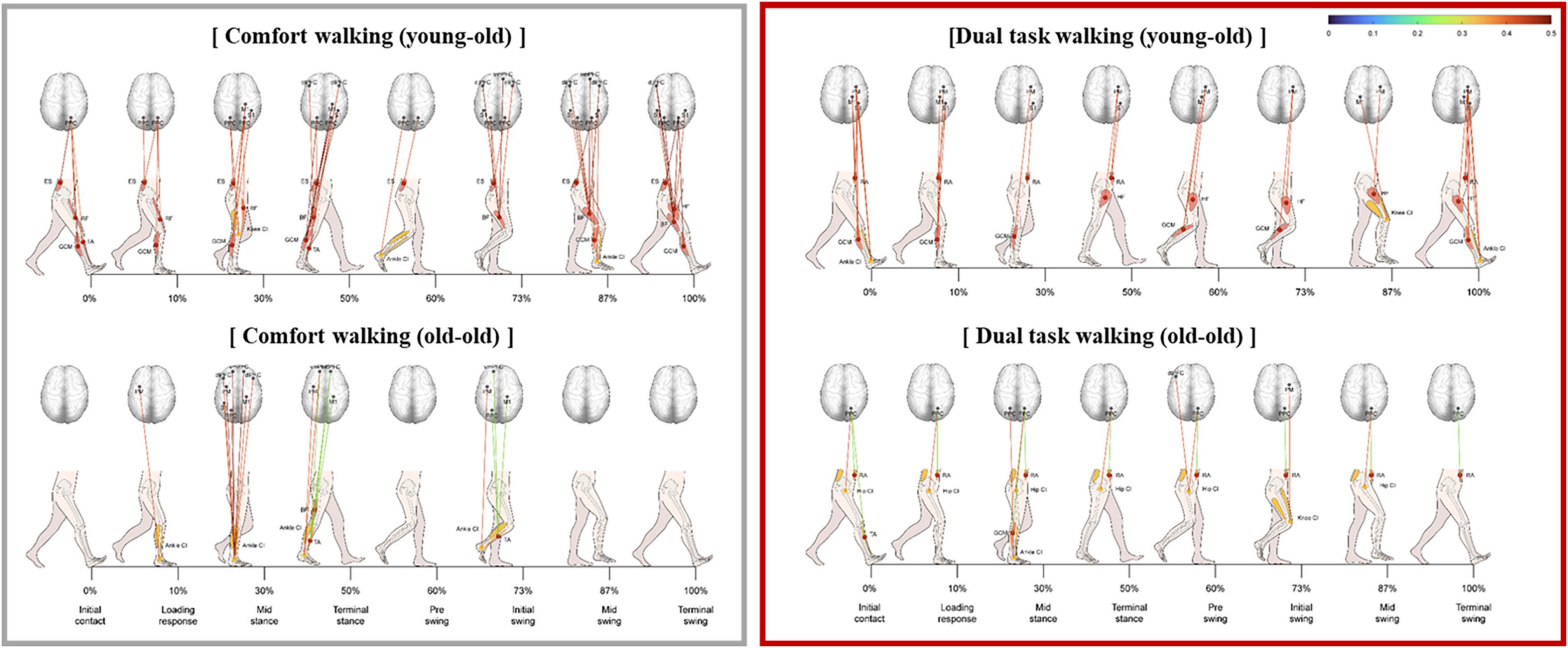
Significant correlation between lower limb muscle activity and cortical activation during cognitive dual-task walking in the young-old and old-old groups.

The significant correlation between lower limb muscle activity and cortical activation was weaker during cognitive dual-task walking than during comfortable walking in both groups (Figure 5). The correlation was weaker during cognitive dual-task walking compared with comfortable walking.

## 4. Discussion

In this study, we investigated the relationships between lower limb muscle activity and cortical activation during treadmill walking in young-old and old-old participants. In the old-old group, whose gait function was more deteriorated than that in the young-old group, mGCM muscle activity and cortical activation showed larger increases than in the young-old group during treadmill walking. In addition, muscle activity correlated strongly with cortical activation in the young-old group, whereas a significant but weaker correlation was observed in the old-old group. This corticomuscular connectivity was influenced by gait speed and addition of a cognitive task, with speed loading increasing the connection and cognitive challenge weakening it.

A previous study demonstrated significant differences in gait function between young-old and old-old participants divided at the age of 75 years (Ferraro, 1980). The results of this study also show significant differences in gait and balance between those two groups. The old-old group was slower and had poorer physical performance than the young-old group, which is consistent with acceleration of decreasing mobility with age (Forrest et al., 2006). Furthermore, the age-related decreases in dual-task cost and gait performance in the old-old group were greater than those of the young-old group under high-attention load conditions (i.e., walking under dual-task conditions; Pothier et al., 2015). Therefore, age-related changes in corticomuscular connectivity between elderly people in different age groups should be investigated further.

During comfortable walking, the old-old group had greater activity of the GCM in the initial contact and initial swing phases than the young-old group. However, ES muscle activity during the terminal swing phase decreased significantly in the old-old group. Elderly people compensate for their reduced physical performance by changing their gait patterns to prevent falls (Maki, 1997). Those changes in gait patterns are particularly evident in the characteristics of muscle activity (Mickelborough et al., 2004). Activation of the TA and soleus muscles in elderly adults is greater than that in young adults during the loading response and mid-stance phases (Schmitz et al., 2009). GCM activity also increased in the old-old group, and coactivation of the ankle joint muscle significantly increased in the initial contact and swing phases. In a previous study, such coactivation in elderly people was significantly higher than that among young and middle-aged subjects while walking (Lee et al., 2017). Muscle coactivation correlated significantly with gait speed in elderly participants, suggesting that the slow gait speed in the old-old group is associated with increased coactivation of the ankle joint muscles.

During comfortable walking, the overall oxyHb concentration within the measured cortical areas was higher in the old-old group than in the young-old group. These results are consistent with the cognitive resource theory of aging that brain activation increases during comfortable walking with age (Beurskens and Bock, 2013). Similarly, older subjects exhibit greater neural activation (hyperactivation) than younger individuals when they perform exercise-related tasks (Ward, 2006). These findings of hyperactivation of the cerebral cortex among old-old people during walking could reflect their more pronounced gait degradation compared with young-old people. Additionally, elderly with cognitive decline due to dementia demonstrated higher oxyHb concentration during walking than did a healthy group (Teo et al., 2021). It was hypothesized that increased cortical activity during walking in people with cognitive decline may represent some form of neural compensation, as suggested by the compensation-related utilization of neural circuits hypothesis (CRUNCH) (Reuter-Lorenz and Cappell, 2008). Since the K-MMSE score is lower in the old-old than in the young-old, hyperactivation in the old-old can be considered as compensation for cognitive decline.

Hyperactivation of the cerebral cortex in the old-old may be associated with depressive symptoms. The GDS-SF score of the old-old was significantly higher than that of the young-old. Functional neuroimaging studies of depressed patients report abnormal activity in the left prefrontal and prefrontal cortex compared to healthy subjects (Okada et al., 2003). A previous study demonstrated increased cortical activity in a depressed group during a working memory task (Matsuo et al., 2007; Fitzgerald et al., 2008). This indicates that hyperactivation of cortical activity due to depressive symptoms is evident in high-demand cognitive environments or task demands (Colich et al., 2017). As the average GDS-SF score of the old-old was 4.85 ± 4.21 (range: 0 ~ 13), which is lower than the cut-off score of 9 (Laudisio et al., 2018), the depressive symptoms in the old-old might be related to hyperactivation.

In this study, lower limb muscle activity correlated with cortical activation among all our elderly participants during comfortable treadmill walking. In recent EEG and EMG studies, coherence and directional analyzes have investigated whether the motor cortex contributes to muscle activity (Winslow et al., 2016; Jensen et al., 2019). In those reports, coherence between the cortical motor region and the TA and BF was enhanced in the swing phase, and the TA had higher connectivity than the BF. In this study, the oxyHb concentration in the cerebral cortex was measured *via* fNIRS, and its correlation with muscle activity was analyzed. Our results support those previous reports showing a close connection between cortical and muscle activity, as measured by EEG–EMG coherence.

The correlation between lower limb muscle activity and cortical activation during comfortable walking was stronger in the swing phase than in the stance phase and was likely related to the CI of the joint muscles. In the swing phase, elderly people can experience an increase in muscle coactivation to gain ankle and knee joint stability, which increases cortical activity. That increased coactivation reinforces the rigidity of the leg joints in elderly people to help them maintain balance and joint stability and thus prevent falls (Thompson et al., 2018). In other studies, the stance phase of walking has been shown to be necessary to maintain an upright posture under dynamic and challenging conditions (Takakusaki, 2017). Our results are consistent with that conclusion because a specific cortical region is involved in muscle activation. In a previous EMG study of reciprocal inhibition during walking, the swing phase required greater control of cortical activation than the other gait phases (Lavoie et al., 1997). In a transcranial magnetic stimulation study using lower extremity muscle motor-evoked potentials, the corticospinal tract was closely linked to muscles that control ankle flexion/extension during the swing phase of the gait cycle (Takahashi et al., 2014).

The correlation between lower limb muscle activity and cortical activation during gait likely weakened in the old-old group, and their gait function decreased compared with that of the young-old group. These findings suggest that old-old people experience greater cortical activation than younger people while walking, but the correlation between cortical activation and muscle activity decreases. The gait control mechanism of the cranial nervous system is also affected by aging, and the use of indirect motor pathways from the PMC, SMA, and PFC increases to maintain gait control despite aging-related functional decline (Herold et al., 2018). In other words, decreased walking ability due to aging requires involvement of secondary motor areas, the PFC, and brain areas other than the primary motor cortex during walking; as a result, the association between cortical activity and muscle activity is reduced.

When walking speed increased, cortical activity decreased. Several studies have reported increased activity in the PFC when walking speed increases, but those studies compared a slow walking speed (1.5 km/h) with a normal walking speed (3.0 km/h; Kim et al., 2017). Thus, comparisons of cortical activity at increased speeds compared to slower speeds can be controversial. In this study, a decrease in cortical activity was observed when walking at a speed of 130% compared to self-selected walking speed. A previous study reported decrease in PFC and M1 activity when elderly participants walked at fast speed compared to slow speed (Herold et al., 2018). In contrast, activity of the subcortical and spinal structures that govern automatic movement increase during fast speed walking (Nordin et al., 2019). Based on these findings and results of this study, cortical activities in the motor and frontal cortical regions are reduced and lower limb muscle activities are increased during fast walking compared to comfortable walking, but the correlation between these two is strengthened, which supports the different roles of cortical activity in speed walking.

This study has some limitations. First, tasks were conducted on a treadmill, which could limit the generalizability of our results. Several studies have reported differences in gait performance and cortical activity between a treadmill and overground walking (Roeder et al., 2018). In our study, we used a treadmill to position the fNIRS device close to the subjects during walking. In future studies, cortical activity should be measured during overground walking in various environments. Second, the correlation between muscle and cortical activities does not imply a direct connection. In an EEG–EMG corticomuscular coherence study, the cortex modulated lower muscle movement in the beta band, including 20 Hz. However, the sampling rate of fNIRS used in the present study was up to only 10 Hz; consequently, the direct connection in the beta band could not be confirmed. Therefore, the correlation between the oxyHb concentration in the cortex and %MVC in the muscles during walking in elderly people does not indicate a direct connection between the brain and muscles. Third, no control group of healthy young adults was used. In this study, the characteristics of cortical and muscle activities were compared between the young-old and the old-old without comparison with health young adults. To help understand overall aging, comparison with healthy young adults is necessary, and there is a limit to interpreting the differences between two aged groups. Fourth, in fNIRS, changes in cerebral blood flow were measured by setting the maximum source detector separation (SDS) to 30 mm without using short SDS channels. Therefore, the recorded fNIRS signals reflect extra- and intracerebral changes; correction using short SDS signals could have reduced the influence of changes in skin blood flow. Therefore, in future studies, skin blood flow should be omitted using short SDS signal measurement equipment (Scholkmann et al., 2013).

## 5. Conclusion

Results of this study indicated significant correlations between lower limb muscle activity and cortical activation during walking, which differed by age group of the elderly. These correlations were also modulated by additional load of gait speed or cognitive dual task. These results suggest that gait function is compensated by regulating corticomotor correlation as well as brain activity during walking in the elderly. We believe that this study deepens understanding of the central-to-peripheral gait control mechanism in elderly people and provides data useful for developing specific gait training targets to prevent falls and to develop a gait intervention program using a robotic device or brain–computer interface for the elderly.

## Data availability statement

The raw data supporting the conclusions of this article will be made available by the authors, without undue reservation.

## Ethics statement

The studies involving human participants were reviewed and approved by Institutional Review Board of Samsung Medical Center (2020–09-172). The patients/participants provided their written informed consent to participate in this study.

## Author contributions

JK, JL, and Y-HK designed the experiment. JK and GL collected the data. JK performed data and statistical analysis with assistance on approach and interpretation from JL, GL, WC, M-HK, W-KY, G-HR, and Y-HK and wrote the manuscript. Y-HK critically evaluated the manuscript. All authors contributed to the article and approved the submitted version.

## Funding

This study was supported by a National Research Foundation of Korea (NRF) grant funded by the Korean government (NRF-2020R1A2C3010304); the MSIT (Ministry of Science and ICT), Korea, under the ICT Creative Consilience program (IITP-2021-2020-0-01821) supervised by the IITP (Institute for Information & Communications Technology Planning & Evaluation); and a Korean Medical Device Development Fund grant funded by the Korean government (MSIT, Ministry of Trade, Industry and Energy, Ministry of Health & Welfare, Ministry of Food and Drug Safety; Project Number: KMDF-RS-2022-00140478).

## Conflict of interest

JK was employed by Ybrain Inc.

The remaining authors declare that the research was conducted in the absence of any commercial or financial relationships that could be construed as a potential conflict of interest.

## Publisher’s note

All claims expressed in this article are solely those of the authors and do not necessarily represent those of their affiliated organizations, or those of the publisher, the editors and the reviewers. Any product that may be evaluated in this article, or claim that may be made by its manufacturer, is not guaranteed or endorsed by the publisher.

## Supplementary material

The Supplementary material for this article can be found online at: https://www.frontiersin.org/articles/10.3389/fnagi.2022.1059563/full#supplementary-material
