# Supplementary material for: Relationship between lower limb muscle activity and cortical activation among elderly people during walking: Effects of fast speed and cognitive dual task
Source: Front Aging Neurosci. 2023 Jan 10;14:1059563. doi: 10.3389/fnagi.2022.1059563 (PMC9871491; doi:10.3389/fnagi.2022.1059563)
Supplement: Supplementary file 1 [file Data_Sheet_1.DOCX]

Supplementary Material

Supplementary Table 1. Lower limb muscle activity in the young-old and old-old groups during comfortable walking

| Gait cycle | Muscle | Total  (*n* = 62) | Young-old  (*n* = 31) | Old-old  (*n* = 31) | *p*-value |
| --- | --- | --- | --- | --- | --- |
| **Initial contact** | Rectus abdominus | 18.6±16.4 | 19.9±17.7 | 17.2±15.2 | 0.512 |
| **(0-2%)** | Erector spine | 25.7±16.8 | 29.5±18.7 | 21.8±13.7 | 0.068 |
|  | Hip flexor | 19.9±12.9 | 20.5±11.1 | 19.3±14.6 | 0.728 |
|  | Rectus femoris | 29.0±12.6 | 28.1±10.6 | 29.9±14.4 | 0.585 |
|  | Biceps femoris | 32.9±15.0 | 33.2±16.5 | 32.7±13.5 | 0.905 |
|  | Tibialis anterior | 33.0±15.1 | 34.8±13.3 | 31.3±16.8 | 0.368 |
|  | **Gastrocnemius** | **16.8±14.2** | **13.2±10.6** | **20.4±16.5** | **0.047^*^** |
| Loading response | Rectus abdominus | 19.2±17.2 | 21.1±18.7 | 17.4±15.7 | 0.409 |
| (2-12%) | Erector spine | 25.1±17.3 | 28.5±19.4 | 21.7±14.5 | 0.124 |
|  | Hip flexor | 23.2±14.6 | 24.0±12.9 | 22.4±16.2 | 0.685 |
|  | Rectus femoris | 32.1±14.4 | 31.7±12.3 | 32.6±16.3 | 0.813 |
|  | Biceps femoris | 32.4±14.9 | 32.1±15.6 | 32.6±14.4 | 0.901 |
|  | Tibialis anterior | 31.2±14.9 | 32.7±13.3 | 29.7±16.4 | 0.425 |
|  | Gastrocnemius | 18.2±14.2 | 14.7±9.8 | 21.7±16.9 | 0.053 |
| Mid-stance | Rectus abdominus | 20.0±17.3 | 22.2±19.4 | 17.9±15.1 | 0.329 |
| (12-31%) | Erector spine | 21.6±15.6 | 23.6±16.8 | 19.6±14.2 | 0.316 |
|  | Hip flexor | 25.0±15.4 | 28.2±16.8 | 24.4±18.0 | 0.548 |
|  | Rectus femoris | 28.7±14.3 | 27.0±10.8 | 30.4±17.1 | 0.354 |
|  | Biceps femoris | 28.4±14.1 | 28.8±13.5 | 28.1±15.0 | 0.843 |
|  | Tibialis anterior | 25.0±13.9 | 25.1±11.9 | 24.9±15.9 | 0.961 |
|  | Gastrocnemius | 28.0±13.1 | 28.4±12.2 | 27.6±14.1 | 0.820 |
| Terminal stance | Rectus abdominus | 19.2±15.9 | 21.6±17.9 | 16.7±13.4 | 0.233 |
| (31-50%) | Erector spine | 21.6±14.4 | 23.9±15.6 | 19.3±12.8 | 0.206 |
|  | Hip flexor | 21.8±14.0 | 22.8±13.5 | 20.8±14.6 | 0.582 |
|  | Rectus femoris | 21.0±12.5 | 19.4±9.3 | 22.7±15.1 | 0.306 |
|  | Biceps femoris | 22.8±12.7 | 23.8±11.8 | 21.8±13.7 | 0.538 |
|  | Tibialis anterior | 23.2±14.2 | 22.7±11.5 | 23.7±16.6 | 0.771 |
|  | Gastrocnemius | 38.2±14.5 | 39.9±14.1 | 36.6±14.9 | 0.376 |

Values represent means ± standard deviations, * bold font, significant difference between the young-old and old-old groups (p < 0.05)

Supplementary Table 1. Lower limb muscle activity in the young-old and old-old groups during comfortable walking (continued)

| Gait cycle | Muscle | Total  (*n* = 62) | Young-old  (*n* = 31) | Old-old  (*n* = 31) | *p*-value |
| --- | --- | --- | --- | --- | --- |
| Pre-swing | Rectus abdominus | 19.8±16.9 | 22.8±19.3 | 16.9±13.6 | 0.173 |
| (50-62%) | Erector spine | 24.3±14.6 | 27.5±16.2 | 21.2±12.3 | 0.092 |
|  | Hip flexor | 23.0±15.0 | 22.9±13.8 | 23.1±16.3 | 0.969 |
|  | Rectus femoris | 19.4±11.9 | 17.7±8.5 | 21.1±14.4 | 0.255 |
|  | Biceps femoris | 21.4±12.5 | 21.9±11.0 | 20.9±14.0 | 0.766 |
|  | Tibialis anterior | 27.0±14.6 | 26.8±12.1 | 25.5±17.7 | 0.882 |
|  | Gastrocnemius | 26.4±14.2 | 23.5±10.6 | 29.2±16.7 | 0.117 |
| **Initial swing** | Rectus abdominus | 20.6±16.7 | 24.0±19.1 | 17.2±13.4 | 0.116 |
| **(62-75%)** | Erector spine | 24.3±15.8 | 27.4±17.9 | 21.2±12.9 | 0.127 |
|  | Hip flexor | 27.0±18.5 | 26.6±16.8 | 27.4±20.4 | 0.863 |
|  | Rectus femoris | 19.8±11.5 | 18.2±9.6 | 21.3±13.2 | 0.304 |
|  | Biceps femoris | 23.3±13.3 | 23.6±12.7 | 22.9±14.0 | 0.836 |
|  | Tibialis anterior | 31.7±14.9 | 32.2±12.2 | 31.2±17.3 | 0.784 |
|  | **Gastrocnemius** | **18.5±13.5** | **14.9±9.9** | **22.2±15.7** | **0.033*** |
| Mid-swing | Rectus abdominus | 20.2±16.7 | 23.3±19.1 | 17.1±13.5 | 0.146 |
| (75-87%) | Erector spine | 24.6±16.3 | 28.0±17.6 | 21.3±14.2 | 0.102 |
|  | Hip flexor | 22.2±15.0 | 22.2±13.1 | 22.2±16.9 | 0.994 |
|  | Rectus femoris | 19.4±10.5 | 17.7±8.5 | 21.0±12.1 | 0.229 |
|  | Biceps femoris | 25.8±13.0 | 26.0±13.6 | 25.7±12.7 | 0.923 |
|  | Tibialis anterior | 30.2±14.2 | 30.9±12.2 | 29.5±16.1 | 0.702 |
|  | Gastrocnemius | 16.8±14.2 | 13.9±11.9 | 19.7±15.8 | 0.111 |
| **Terminal swing** | Rectus abdominus | 19.2±16.8 | 21.2±18.7 | 17.2±14.7 | 0.355 |
| **(87-100%)** | **Erector spine** | **25.2±15.9** | **29.2±18.0** | **21.2±12.5** | **0.046*** |
|  | Hip flexor | 18.8±13.5 | 19.3±11.4 | 18.3±15.5 | 0.785 |
|  | Rectus femoris | 23.6±10.8 | 21.9±8.1 | 25.4±12.8 | 0.206 |
|  | Biceps femoris | 31.0±14.0 | 31.6±15.9 | 30.4±12.0 | 0.750 |
|  | Tibialis anterior | 31.4±14.3 | 32.3±12.4 | 30.5±16.2 | 0.625 |
|  | Gastrocnemius | 16.7±14.3 | 13.5±11.1 | 19.8±16.5 | 0.086 |

Values represent means ± standard deviations, * bold font, significant difference between the young-old and old-old groups (p < 0.05)

Supplementary Table 2. Lower limb muscle coactivation in the young-old and old-old groups during comfortable walking

| Gait cycle | Joint (muscle pair) | Total  (*n* = 62) | Young-old  (*n* = 31) | Old-old  (*n* = 31) | *p*-value |
| --- | --- | --- | --- | --- | --- |
| **Initial contact** | Hip (RA:ES) | 113.4±38.9 | 120.4±46.7 | 115.8±33.4 | 0.650 |
| **(0-2%)** | Knee (RF:BF) | 106.6±20.6 | 102.6±21.3 | 110.9±19.1 | 0.116 |
|  | **Ankle (TA:mGCM)** | **64.0±26.8** | **51.8±20.4** | **77.0±26.9** | **<0.001*** |
| **Loading response** | Hip (RA:ES) | 115.5±41.1 | 116.8±46.6 | 114.2±35.7 | 0.804 |
| **(2-12%)** | Knee (RF:BF) | 99.5±24.0 | 98.0±23.6 | 101.1±24.6 | 0.614 |
|  | **Ankle (TA:mGCM)** | **69.9±28.7** | **60.5±24.8** | **79.4±29.6** | **0.008*** |
| Mid-stance | Hip (RA:ES) | 107.1±41.6 | 107.3±47.1 | 107.0±36.2 | 0.980 |
| (12-31%) | Knee (RF:BF) | 97.9±23.3 | 99.5±21.6 | 96.2±25.1 | 0.590 |
|  | Ankle (TA:mGCM) | 108.7±24.6 | 107.9±24.8 | 109.4±24.8 | 0.804 |
| Terminal stance | Hip (RA:ES) | 109.2±38.8 | 108.7±46.5 | 109.9±30.2 | 0.904 |
| (31-50%) | Knee (RF:BF) | 102.3±25.4 | 105.6±25.3 | 99.0±25.4 | 0.312 |
|  | Ankle (TA:mGCM) | 128.6±24.7 | 129.4±22.8 | 127.8±26.8 | 0.804 |
| Pre-swing | Hip (RA:ES) | 113.7±38.1 | 113.0±45.0 | 114.4±30.5 | 0.883 |
| (50-62%) | Knee (RF:BF) | 103.5±26.6 | 106.7±27.1 | 100.4±26.2 | 0.361 |
|  | Ankle (TA:mGCM) | 100.2±27.0 | 95.7±25.6 | 104.7±28.1 | 0.190 |
| **Initial swing** | Hip (RA:ES) | 110.6±40.3 | 109.2±47.2 | 112.1±32.8 | 0.779 |
| **(62-75%)** | Knee (RF:BF) | 107.3±27.2 | 109.8±27.2 | 104.9±27.3 | 0.481 |
|  | **Ankle (TA:mGCM)** | **71.4±26.5** | **61.5±21.1** | **81.2±27.9** | **0.003*** |
| **Mid-swing** | Hip (RA:ES) | 112.2±40.9 | 112.0±48.4 | 112.6±32.6 | 0.956 |
| **(75-87%)** | Knee (RF:BF) | 114.5±22.8 | 116.6±24.1 | 112.4±21.5 | 0.476 |
|  | **Ankle (TA:mGCM)** | **66.1±25.0** | **56.7±21.4** | **75.5±25.1** | **0.002*** |
| **Terminal swing** | Hip (RA:ES) | 116.6±40.8 | 118.1±48.1 | 115.2±32.7 | 0.787 |
| **(87-100%)** | Knee (RF:BF) | 112.7±22.7 | 114.2±23.9 | 111.1±21.8 | 0.603 |
|  | **Ankle (TA:mGCM)** | **64.2±25.3** | **55.6±20.3** | **72.7±27.1** | **0.007*** |

Values represent means ± standard deviations, RA, rectus abdominus; ES, erector spine; RF, rectus femoris; BF, biceps femoris; TA, tibialis anterior; mGCM, gastrocnemius. * bold font, significant difference between the young-old and old-old groups (p < 0.05)

Supplementary Table 3. Integral values of relative oxyHb in the young-old and old-old groups during comfortable walking

| Region of interest | | Ch. | Total  (*n* = 62) | Young-old  (*n* = 31) | Old-old  (*n* = 31) | *p*-value |
| --- | --- | --- | --- | --- | --- | --- |
| M1-leg | Lt. | Ch.61 | 0.078±0.099 | 0.071±0.098 | 0.086±0.101 | 0.547 |
|  | Rt. | Ch.60 | 0.090±0.103 | 0.075±0.103 | 0.105±0.102 | 0.256 |
| **PM** | Lt. | Ch.41 | 0.108±0.168 | 0.103±0.189 | 0.113±0.147 | 0.824 |
|  |  | Ch.42 | 0.050±0.121 | 0.038±0.111 | 0.062±0.132 | 0.451 |
|  | Rt. | Ch.33 | 0.046±0.115 | 0.032±0.102 | 0.060±0.126 | 0.343 |
|  |  | **Ch.34** | **0.082±0.173** | **0.039±0.157** | **0.125±0.181** | **0.052** |
| SMA |  | Ch.9 | 0.061±0.135 | 0.047±0.108 | 0.075±0.158 | 0.431 |
|  |  | Ch.11 | 0.053±0.127 | 0.034±0.109 | 0.072±0.142 | 0.244 |
| S1 | Lt. | Ch.44 | 0.106±0.129 | 0.096±0.131 | 0.116±0.129 | 0.543 |
|  |  | Ch.46 | 0.074±0.103 | 0.060±0.111 | 0.087±0.094 | 0.306 |
|  | Rt. | Ch.37 | 0.099±0.116 | 0.090±0.118 | 0.109±0.114 | 0.528 |
|  |  | Ch.38 | 0.060±0.089 | 0.049±0.090 | 0.071±0.089 | 0.339 |
| PPC | Lt. | Ch.65 | 0.061±0.121 | 0.060±0.127 | 0.062±0.116 | 0.940 |
|  |  | Ch.68 | 0.059±0.115 | 0.044±0.106 | 0.075±0.123 | 0.292 |
|  | Rt. | Ch.66 | 0.090±0.127 | 0.082±0.145 | 0.099±0.107 | 0.595 |
|  |  | Ch.70 | 0.026±0.193 | 0.008±0.171 | 0.043±0.215 | 0.486 |
| dlPFC | Lt. | Ch.2 | 0.049±0.128 | 0.035±0.105 | 0.064±0.148 | 0.384 |
|  |  | Ch.6 | 0.040±0.086 | 0.028±0.069 | 0.052±0.100 | 0.271 |
|  | Rt. | Ch.17 | 0.034±0.073 | 0.028±0.070 | 0.040±0.076 | 0.513 |
|  |  | Ch.19 | 0.041±0.107 | 0.026±0.098 | 0.056±0.115 | 0.265 |
| vmPFC | Lt. | Ch.5 | 0.115±0.150 | 0.108±0.126 | 0.122±0.174 | 0.727 |
|  |  | Ch.13 | 0.094±0.159 | 0.100±0.169 | 0.089±0.151 | 0.790 |
|  | Rt. | Ch.15 | 0.109±0.215 | 0.090±0.140 | 0.127±0.273 | 0.489 |
|  |  | Ch.22 | 0.087±0.113 | 0.075±0.116 | 0.100±0.111 | 0.396 |

Values represent means ± standard deviations, M1-leg, primary leg motor cortex; PM, premotor area; SMA, supplementary motor area; S1, primary somatosensory cortex; PPC, posterior parietal cortex; dlPFC, dorsolateral prefrontal cortex; vmPFC, ventromedial prefrontal cortex. * bold font, significant difference between the young-old and old-old groups (p < 0.05)

Supplementary Table 4. Lower limb muscle activity in all the participants during fast walking compared with comfortable walking

| Gait cycle | Muscle | Comfortable walking | Fast walking | *p*-value |
| --- | --- | --- | --- | --- |
| **Initial contact** | Rectus abdominus | 18.6±16.4 | 20.7±17.9 | 0.182 |
| **(0-2%)** | Erector spine | 25.7±16.8 | 27.6±16.0 | 0.287 |
|  | **Hip flexor** | **19.9±12.9** | **23.3±14.2** | **0.014*** |
|  | **Rectus femoris** | **29.0±12.6** | **32.4±14.2** | **0.016*** |
|  | **Biceps femoris** | **32.9±15.0** | **39.0±15.8** | **<0.001*** |
|  | **Tibialis anterior** | **33.0±15.1** | **38.0±17.2** | **0.005*** |
|  | **Gastrocnemius** | **16.8±14.2** | **20.6±15.9** | **0.011*** |
| **Loading response** | Rectus abdominus | 19.2±17.2 | 21.6±18.1 | 0.138 |
| **(2-12%)** | Erector spine | 25.1±17.3 | 28.3±15.8 | 0.072 |
|  | **Hip flexor** | **23.2±14.6** | **26.6±14.5** | **0.031*** |
|  | **Rectus femoris** | **32.1±14.4** | **36.0±16.2** | **0.017*** |
|  | **Biceps femoris** | **32.4±14.9** | **37.5±14.8** | **0.001*** |
|  | **Tibialis anterior** | **31.2±14.9** | **35.3±15.8** | **0.012*** |
|  | **Gastrocnemius** | **18.2±14.2** | **23.6±16.6** | **<0.001*** |
| **Mid-stance** | **Rectus abdominus** | **20.0±17.3** | **23.1±18.8** | **0.040*** |
| **(12-31%)** | Erector spine | 21.6±15.6 | 23.7±15.5 | 0.262 |
|  | **Hip flexor** | **25.0±15.4** | **29.0±14.4** | **0.015*** |
|  | Rectus femoris | 28.7±14.3 | 30.2±13.5 | 0.343 |
|  | **Biceps femoris** | **28.4±14.1** | **32.1±13.9** | **0.007*** |
|  | Tibialis anterior | 25.0±13.9 | 27.5±15.3 | 0.053 |
|  | **Gastrocnemius** | **28.0±13.1** | **35.4±17.1** | **<0.001*** |
| **Terminal stance** | Rectus abdominus | 19.2±15.9 | 21.2±17.5 | 0.220 |
| **(31-50%)** | Erector spine | 21.6±14.4 | 24.1±14.4 | 0.140 |
|  | **Hip flexor** | **21.8±14.0** | **25.1±12.9** | **0.040*** |
|  | Rectus femoris | 21.0±12.5 | 22.3±13.2 | 0.294 |
|  | Biceps femoris | 22.8±12.7 | 24.5±14.0 | 0.242 |
|  | Tibialis anterior | 23.2±14.2 | 25.3±16.3 | 0.171 |
|  | **Gastrocnemius** | **38.2±14.5** | **43.5±18.7** | **0.029*** |

Values represent means ± standard deviations, * bold font, significant difference between fast walking and comfortable walking (p < 0.05)

Supplementary Table 4. Lower limb muscle activity in all the participants during fast walking compared with comfortable walking (continued)

| Gait cycle | Muscle | Comfortable walking | Fast walking | *p*-value |
| --- | --- | --- | --- | --- |
| **Pre-swing** | **Rectus abdominus** | **19.8±16.9** | **23.1±18.2** | **0.047*** |
| **(50-62%)** | Erector spine | 24.3±14.6 | 27.3±15.2 | 0.081 |
|  | Hip flexor | 23.0±15.0 | 25.7±12.8 | 0.107 |
|  | Rectus femoris | 19.4±11.9 | 25.7±12.8 | 0.248 |
|  | **Biceps femoris** | **21.4±12.5** | **25.1±14.5** | **0.015*** |
|  | Tibialis anterior | 27.0±14.6 | 29.7±16.9 | 0.095 |
|  | Gastrocnemius | 26.4±14.2 | 29.2±15.5 | 0.089 |
| **Initial swing** | **Rectus abdominus** | **20.6±16.7** | **23.6±18.3** | **0.039*** |
| **(62-75%)** | Erector spine | 24.3±15.8 | 26.1±14.2 | 0.296 |
|  | Hip flexor | 27.0±18.5 | 29.8±15.5 | 0.176 |
|  | Rectus femoris | 19.8±11.5 | 22.4±14.0 | 0.083 |
|  | Biceps femoris | 23.3±13.3 | 25.1±14.5 | 0.209 |
|  | Tibialis anterior | 31.7±14.9 | 34.8±16.3 | 0.052 |
|  | Gastrocnemius | 18.5±13.5 | 20.4±14.4 | 0.207 |
| **Mid-swing** | Rectus abdominus | 20.2±16.7 | 20.9±17.1 | 0.771 |
| **(75-87%)** | Erector spine | 24.6±16.3 | 25.9±14.3 | 0.427 |
|  | **Hip flexor** | **22.2±15.0** | **26.3±14.5** | **0.007*** |
|  | Rectus femoris | 19.4±10.5 | 21.8±12.3 | 0.067 |
|  | **Biceps femoris** | **25.8±13.0** | **29.8±12.7** | **0.009*** |
|  | **Tibialis anterior** | **30.2±14.2** | **32.9±14.9** | **0.032*** |
|  | Gastrocnemius | 16.8±14.2 | 18.5±15.5 | 0.338 |
| **Terminal swing** | Rectus abdominus | 19.2±16.8 | 19.8±16.7 | 0.820 |
| **(87-100%)** | Erector spine | 25.2±15.9 | 27.5±15.4 | 0.160 |
|  | **Hip flexor** | **18.8±13.5** | **22.1±14.1** | **0.019*** |
|  | **Rectus femoris** | **23.6±10.8** | **26.2±14.2** | **0.018*** |
|  | **Biceps femoris** | **31.0±14.0** | **36.8±14.2** | **0.001*** |
|  | **Tibialis anterior** | **31.4±14.3** | **35.4±14.8** | **0.005*** |
|  | Gastrocnemius | 16.7±14.3 | 19.4±15.8 | 0.107 |

Values represent means ± standard deviations, * bold font, significant difference between fast walking and comfortable walking (p < 0.05)

Supplementary Table 5. Lower limb muscle coactivation in all the participants during fast walking compared with comfortable walking

| Gait cycle | Joint (muscle pair) | Comfortable walking | Fast walking | *p*-value |
| --- | --- | --- | --- | --- |
| Initial contact (0-2%) | Hip (RA:ES) | 113.4±38.9 | 118.2±38.3 | 0.744 |
|  | Knee (RF:BF) | 106.6±20.6 | 108.0±22.6 | 0.216 |
|  | Ankle (TA:mGCM) | 64.0±26.8 | 67.8±28.6 | 0.146 |
| **Loading response (2-12%)** | Hip (RA:ES) | 115.5±41.1 | 116.2±39.1 | 0.593 |
|  | Knee (RF:BF) | 99.5±24.0 | 101.6±23.1 | 0.374 |
|  | **Ankle (TA:mGCM)** | **69.9±28.7** | **76.9±28.9** | **0.025*** |
| **Mid-stance (12-31%)** | Hip (RA:ES) | 107.1±41.6 | 105.6±41.3 | 0.907 |
|  | Knee (RF:BF) | 97.9±23.3 | 101.0±22.8 | 0.216 |
|  | **Ankle (TA:mGCM)** | **108.7±24.6** | **115.2±23.1** | **0.001*** |
| Terminal-stance (31-50%) | Hip (RA:ES) | 109.2±38.8 | 110.5±38.9 | 0.502 |
|  | Knee (RF:BF) | 102.3±25.4 | 102.5±24.3 | 0.835 |
|  | Ankle (TA:mGCM) | 128.6±24.7 | 130.4±23.5 | 0.338 |
| Pre-swing (50-62%) | Hip (RA:ES) | 113.7±38.1 | 111.9±37.3 | 0.867 |
|  | Knee (RF:BF) | 103.5±26.6 | 100.2±24.0 | 0.230 |
|  | Ankle (TA:mGCM) | 100.2±27.0 | 100.4±23.7 | 0.911 |
| Initial swing (62-75%) | Hip (RA:ES) | 110.6±40.3 | 109.0±37.9 | 0.892 |
|  | Knee (RF:BF) | 107.3±27.2 | 105.9±23.2 | 0.723 |
|  | Ankle (TA:mGCM) | 71.4±26.5 | 71.6±23.2 | 0.897 |
| Mid-swing (75-87%) | Hip (RA:ES) | 112.2±40.9 | 113.7±38.3 | 0.498 |
|  | Knee (RF:BF) | 114.5±22.8 | 116.6±21.7 | 0.313 |
|  | Ankle (TA:mGCM) | 66.1±25.0 | 67.3±23.3 | 0.727 |
| Terminal swing (87-100%) | Hip (RA:ES) | 116.6±40.8 | 119.3±38.1 | 0.293 |
|  | Knee (RF:BF) | 112.7±22.7 | 115.8±22.6 | 0.170 |
|  | Ankle (TA:mGCM) | 64.2±25.3 | 66.4±26.3 | 0.493 |

Values represent means ± standard deviations, RA, rectus abdominus; ES, erector spine; RF, rectus femoris; BF, biceps femoris; TA, tibialis anterior; mGCM, gastrocnemius. * bold font, significant difference between fast walking and comfortable walking (p < 0.05)

Supplementary Table 6. Integral values of relative oxyHb in all the participants during fast walking

| Region of interest | | Ch. | Comfortable walking | Fast walking | p-value |
| --- | --- | --- | --- | --- | --- |
| M1-leg | Lt. | Ch.61 | 0.078±0.099 | 0.075±0.115 | 0.667 |
|  | Rt. | Ch.60 | 0.090±0.103 | 0.089±0.114 | 0.928 |
| PM | Lt. | Ch.41 | 0.108±0.168 | 0.087±0.169 | 0.162 |
|  |  | Ch.42 | 0.050±0.121 | 0.057±0.152 | 0.665 |
|  | Rt. | Ch.33 | 0.046±0.115 | 0.029±0.117 | 0.094 |
|  |  | Ch.34 | 0.082±0.173 | 0.084±0.146 | 0.580 |
| **SMA** |  | **Ch.9** | **0.061±0.135** | **0.043±0.140** | **0.046*** |
|  |  | Ch.11 | 0.053±0.127 | 0.047±0.129 | 0.461 |
| S1 | Lt. | Ch.44 | 0.106±0.129 | 0.114±0.213 | 0.723 |
|  |  | Ch.46 | 0.074±0.103 | 0.071±0.112 | 0.733 |
|  | Rt. | Ch.37 | 0.099±0.116 | 0.090±0.130 | 0.267 |
|  |  | Ch.38 | 0.060±0.089 | 0.055±0.102 | 0.451 |
| PPC | Lt. | Ch.65 | 0.061±0.121 | 0.057±0.144 | 0.757 |
|  |  | Ch.68 | 0.059±0.115 | 0.037±0.128 | 0.058 |
|  | Rt. | Ch.66 | 0.090±0.127 | 0.070±0.128 | 0.064 |
|  |  | Ch.70 | 0.026±0.193 | 0.043±0.143 | 0.440 |
| dlPFC | Lt. | Ch.2 | 0.049±0.128 | 0.033±0.147 | 0.106 |
|  |  | Ch.6 | 0.040±0.086 | 0.032±0.090 | 0.194 |
|  | Rt. | Ch.17 | 0.034±0.073 | 0.025±0.085 | 0.153 |
|  |  | Ch.19 | 0.041±0.107 | 0.034±0.128 | 0.472 |
| **vmPFC** | **Lt.** | **Ch.5** | **0.115±0.150** | **0.095±0.161** | **0.014*** |
|  |  | Ch.13 | 0.094±0.159 | 0.069±0.181 | 0.088 |
|  | Rt. | Ch.15 | 0.109±0.215 | 0.077±0.163 | 0.332 |
|  |  | Ch.22 | 0.087±0.113 | 0.083±0.149 | 0.708 |

Values represent means ± standard deviations; M1-leg, primary leg motor cortex; PM, premotor area; SMA, supplementary motor area; S1, primary somatosensory cortex; PPC, posterior parietal cortex; dlPFC, dorsolateral prefrontal cortex; vmPFC, ventromedial prefrontal cortex. * bold font, significant difference between fast walking and comfortable walking (p < 0.05)

Supplementary Table 7. Lower limb muscle activity in all the participants during cognitive dual-task walking compared with comfortable walking

| Gait cycle | Muscle | Comfortable walking | Dual-task walking | *p*-value |
| --- | --- | --- | --- | --- |
| **Initial contact** | Rectus abdominus | 18.6±16.4 | 21.0±16.2 | 0.129 |
| **(0-2%)** | Erector spine | 25.7±16.8 | 27.2±15.2 | 0.331 |
|  | Hip flexor | 19.9±12.9 | 19.1±11.7 | 0.503 |
|  | Rectus femoris | 29.0±12.6 | 32.1±15.6 | 0.102 |
|  | **Biceps femoris** | **32.9±15.0** | **33.1±15.2** | **0.022*** |
|  | **Tibialis anterior** | **33.0±15.1** | **35.8±16.9** | **0.047*** |
|  | Gastrocnemius | 16.8±14.2 | 15.8±12.0 | 0.346 |
| **Loading response** | Rectus abdominus | 19.2±17.2 | 21.8±16.9 | 0.115 |
| **(2-12%)** | Erector spine | 25.1±17.3 | 26.5±14.8 | 0.391 |
|  | Hip flexor | 23.2±14.6 | 22.0±13.2 | 0.383 |
|  | Rectus femoris | 32.1±14.4 | 34.9±16.4 | 0.148 |
|  | **Biceps femoris** | **32.4±14.9** | **36.0±16.7** | **0.023*** |
|  | **Tibialis anterior** | **31.2±14.9** | **34.1±16.6** | **0.038*** |
|  | Gastrocnemius | 18.2±14.2 | 17.4±12.9 | 0.456 |
| Mid-stance | Rectus abdominus | 20.0±17.3 | 22.7±17.7 | 0.107 |
| (12-31%) | Erector spine | 21.6±15.6 | 22.2±13.1 | 0.652 |
|  | Hip flexor | 25.0±15.4 | 23.7±13.9 | 0.340 |
|  | Rectus femoris | 28.7±14.3 | 30.6±15.1 | 0.236 |
|  | Biceps femoris | 28.4±14.1 | 30.5±14.6 | 0.101 |
|  | Tibialis anterior | 25.0±13.9 | 26.4±15.5 | 0.267 |
|  | Gastrocnemius | 28.0±13.1 | 26.9±13.0 | 0.471 |
| **Terminal stance** | Rectus abdominus | 19.2±15.9 | 22.2±17.1 | 0.050 |
| **(31-50%)** | Erector spine | 21.6±14.4 | 22.7±13.2 | 0.411 |
|  | Hip flexor | 21.8±14.0 | 20.8±12.4 | 0.413 |
|  | Rectus femoris | 21.0±12.5 | 22.7±13.3 | 0.192 |
|  | Biceps femoris | 22.8±12.7 | 23.9±12.9 | 0.344 |
|  | Tibialis anterior | 23.2±14.2 | 23.2±19.0 | 0.981 |
|  | **Gastrocnemius** | **38.2±14.5** | **32.3±12.8** | **0.003*** |

Values represent means ± standard deviations, * bold font, significant difference between cognitive dual-task walking and comfortable walking (p < 0.05)

Supplementary Table 7. Lower limb muscle activity in all the participants during cognitive dual-task walking compared with comfortable walking (continued)

| Gait cycle | Muscle | Comfortable walking | Dual-task walking | *p*-value |
| --- | --- | --- | --- | --- |
| **Pre-swing** | **Rectus abdominus** | **19.8±16.9** | **23.9±18.1** | **0.015*** |
| **(50-62%)** | Erector spine | 24.3±14.6 | 26.5±14.5 | 0.173 |
|  | Hip flexor | 23.0±15.0 | 22.5±12.8 | 0.739 |
|  | Rectus femoris | 19.4±11.9 | 21.2±13.0 | 0.185 |
|  | Biceps femoris | 21.4±12.5 | 21.9±13.0 | 0.630 |
|  | Tibialis anterior | 27.0±14.6 | 17.1±15.1 | 0.993 |
|  | Gastrocnemius | 26.4±14.2 | 26.2±12.2 | 0.854 |
| **Initial swing** | **Rectus abdominus** | **20.6±16.7** | **24.3±18.1** | **0.024*** |
| **(62-75%)** | Erector spine | 24.3±15.8 | 25.6±13.9 | 0.445 |
|  | Hip flexor | 27.0±18.5 | 26.0±15.7 | 0.597 |
|  | Rectus femoris | 19.8±11.5 | 22.2±13.2 | 0.063 |
|  | Biceps femoris | 23.3±13.3 | 23.9±14.0 | 0.578 |
|  | Tibialis anterior | 31.7±14.9 | 32.1±15.3 | 0.752 |
|  | Gastrocnemius | 18.5±13.5 | 16.9±10.5 | 0.107 |
| **Mid-swing** | Rectus abdominus | 20.2±16.7 | 22.5±16.8 | 0.162 |
| **(75-87%)** | Erector spine | 24.6±16.3 | 25.5±14.3 | 0.589 |
|  | Hip flexor | 22.2±15.0 | 22.2±14.1 | 0.998 |
|  | **Rectus femoris** | **19.4±10.5** | **22.2±13.0** | **0.043*** |
|  | Biceps femoris | 25.8±13.0 | 27.9±14.5 | 0.136 |
|  | Tibialis anterior | 30.2±14.2 | 31.9±15.5 | 0.153 |
|  | **Gastrocnemius** | **16.8±14.2** | **14.5±10.1** | **0.041*** |
| **Terminal swing** | Rectus abdominus | 19.2±16.8 | 21.2±16.3 | 0.231 |
| **(87-100%)** | Erector spine | 25.2±15.9 | 26.9±15.0 | 0.307 |
|  | Hip flexor | 18.8±13.5 | 18.4±13.0 | 0.780 |
|  | Rectus femoris | 23.6±10.8 | 26.4±13.6 | 0.083 |
|  | **Biceps femoris** | **31.0±14.0** | **34.2±16.0** | **0.046*** |
|  | Tibialis anterior | 31.4±14.3 | 33.7±16.4 | 0.096 |
|  | Gastrocnemius | 16.7±14.3 | 14.9±10.9 | 0.129 |

Values represent means ± standard deviations, * bold font, significant difference between cognitive dual-task walking and comfortable walking (p < 0.05)

Supplementary Table 8. Lower limb muscle coactivation in all the participants during cognitive dual-task walking compared with comfortable walking

| Gait cycle | Joint (muscle pair) | Comfortable walking | Dual-task walking | *p*-value |
| --- | --- | --- | --- | --- |
| Initial contact (0-2%) | Hip (RA:ES) | 113.4±38.9 | 115.3±38.3 | 0.370 |
|  | Knee (RF:BF) | 106.6±20.6 | 105.5±21.7 | 0.847 |
|  | Ankle (TA:mGCM) | 64.0±26.8 | 60.5±27.7 | 0.219 |
| Loading response (2-12%) | Hip (RA:ES) | 115.5±41.1 | 112.3±38.8 | 0.299 |
|  | Knee (RF:BF) | 99.5±24.0 | 100.5±21.6 | 0.705 |
|  | Ankle (TA:mGCM) | 69.9±28.7 | 66.2±28.3 | 0.138 |
| Mid-stance (12-31%) | Hip (RA:ES) | 107.1±41.6 | 102.4±39.8 | 0.100 |
|  | Knee (RF:BF) | 97.9±23.3 | 98.4±22.7 | 0.825 |
|  | Ankle (TA:mGCM) | 108.7±24.6 | 104.9±24.4 | 0.138 |
| Terminal stance (31-50%) | Hip (RA:ES) | 109.2±38.8 | 104.6±38.3 | 0.146 |
|  | Knee (RF:BF) | 102.3±25.4 | 100.3±25.7 | 0.320 |
|  | Ankle (TA:mGCM) | 128.6±24.7 | 131.0±25.3 | 0.192 |
| **Pre-swing (50-62%)** | Hip (RA:ES) | 113.7±38.1 | 108.6±38.1 | 0.141 |
|  | **Knee (RF:BF)** | **103.5±26.6** | **99.1±28.0** | **0.044*** |
|  | Ankle (TA:mGCM) | 100.2±27.0 | 101.8±26.1 | 0.493 |
| **Initial swing (62-75%)** | Hip (RA:ES) | 110.6±40.3 | 106.7±38.3 | 0.260 |
|  | **Knee (RF:BF)** | **107.3±27.2** | **102.5±26.4** | **0.036*** |
|  | Ankle (TA:mGCM) | 71.4±26.5 | 69.4±22.8 | 0.384 |
| **Mid-swing (75-87%)** | Hip (RA:ES) | 112.2±40.9 | 110.2±37.6 | 0.567 |
|  | Knee (RF:BF) | 114.5±22.8 | 116.6±21.6 | 0.313 |
|  | **Ankle (TA:mGCM)** | **66.1±25.0** | **61.3±23.7** | **0.009*** |
| Terminal swing (87-100%) | Hip (RA:ES) | 116.6±40.8 | 114.9±38.0 | 0.625 |
|  | Knee (RF:BF) | 112.7±22.7 | 112.2±21.6 | 0.851 |
|  | Ankle (TA:mGCM) | 64.2±25.3 | 60.8±25.5 | 0.115 |

Values represent mean ± standard deviation, RA, rectus abdominus; ES, erector spine; RF, rectus femoris; BF, biceps femoris; TA, tibialis anterior; mGCM, gastrocnemius. * bold font, significant difference between cognitive dual-task walking and comfortable walking (p < 0.05)

Supplementary Table 9. Integral values of relative oxyHb in all the participants during cognitive dual-task walking

| Region of interest | | Ch. | Comfortable walking | Dual-task walking | *p*-value |
| --- | --- | --- | --- | --- | --- |
| **M1-leg** | **Lt.** | **Ch.61** | **0.078±0.099** | **0.100±0.102** | **0.038*** |
|  | **Rt.** | **Ch.60** | **0.090±0.103** | **0.120±0.098** | **0.001*** |
| **PM** | **Lt.** | **Ch.41** | **0.108±0.168** | **0.139±0.171** | **0.012*** |
|  |  | **Ch.42** | **0.050±0.121** | **0.216±0.489** | **0.008*** |
|  | **Rt.** | **Ch.33** | **0.046±0.115** | **0.084±0.165** | **0.011*** |
|  |  | **Ch.34** | **0.082±0.173** | **0.146±0.207** | **0.025*** |
| **SMA** |  | **Ch.9** | **0.061±0.135** | **0.106±0.162** | **<0.001*** |
|  |  | **Ch.11** | **0.053±0.127** | **0.105±0.144** | **<0.001*** |
| **S1** | **Lt.** | Ch.44 | 0.106±0.129 | 0.134±0.137 | 0.064 |
|  |  | **Ch.46** | **0.074±0.103** | **0.110±0.110** | **0.002*** |
|  | Rt. | Ch.37 | 0.099±0.116 | 0.107±0.138 | 0.389 |
|  |  | Ch.38 | 0.060±0.089 | 0.081±0.114 | 0.062 |
| **PPC** | Lt. | Ch.65 | 0.061±0.121 | 0.0880±0.164 | 0.218 |
|  |  | Ch.68 | 0.059±0.115 | 0.088±0.170 | 0.126 |
|  | **Rt.** | **Ch.66** | **0.090±0.127** | **0.138±0.173** | **0.012*** |
|  |  | **Ch.70** | **0.026±0.193** | **0.090±0.202** | **0.030*** |
| **dlPFC** | **Lt.** | **Ch.2** | **0.049±0.128** | **0.085±0.170** | **0.010*** |
|  |  | **Ch.6** | **0.040±0.086** | **0.061±0.100** | **0.007*** |
|  | **Rt.** | **Ch.17** | **0.034±0.073** | **0.066±0.097** | **<0.001*** |
|  |  | **Ch.19** | **0.041±0.107** | **0.096±0.139** | **<0.001*** |
| **vmPFC** | **Lt.** | **Ch.5** | **0.115±0.150** | **0.202±0.180** | **<0.001*** |
|  |  | **Ch.13** | **0.094±0.159** | **0.186±0.201** | **<0.001*** |
|  | **Rt.** | **Ch.15** | **0.109±0.215** | **0.211±0.256** | **<0.001*** |
|  |  | **Ch.22** | **0.087±0.113** | **0.167±0.190** | **<0.001*** |

Values represent means ± standard deviations, M1-leg, primary leg motor cortex; PM, premotor area; SMA, supplementary motor area; S1, primary somatosensory cortex; PPC, posterior parietal cortex; dlPFC, dorsolateral prefrontal cortex; vmPFC, ventromedial prefrontal cortex. * bold font, significant difference between cognitive dual-task walking and comfortable walking (p < 0.05)
